# Dynamics and consequences of differential RNA isoform production during cardiomyocyte fate determination and early-stage maturation

**DOI:** 10.1101/gr.281150.125

**Published:** 2026-05

**Authors:** Joanna Delimata-Raczek, Natalia Koralewska, Bart Krist, Magdalena Rakoczy, Marek Figlerowicz, Ireneusz Stolarek

**Affiliations:** Institute of Bioorganic Chemistry, Polish Academy of Sciences, 61-704 Poznan, Poland

## Abstract

Cellular identity is dictated by precise transcriptomic programs; however, the mechanisms that dynamically shape the transcriptome by producing diverse RNA isoforms remain incompletely understood. Here, through longitudinal transcriptomic profiling of cardiomyogenesis, we delineate pervasive RNA isoform switching across the developmental trajectory of cardiac differentiation. We show that the changes in isoform accumulation are largely decoupled from shifts in overall gene expression levels and arise predominantly from alternative transcription start and termination rather than splicing. This regulatory layer preferentially targets genes essential for cardiac function, specifically those encoding contractile machinery and ion channel complexes. We demonstrate that genes undergoing isoform switching without changing overall expression constitute a functionally distinct cohort, establishing isoform switching as an independent layer of gene regulation. Furthermore, we characterize stage-specific expression dynamics for numerous RNA-binding proteins and link their expression to specific isoform switching events. Our findings support a coordinated mechanism for the targeted regulation of RNA diversity, positioning isoform switching as a primary driver of transcriptomic maturation during lineage commitment.

The directed differentiation of induced pluripotent stem cells (iPSCs) into cardiomyocytes (induced cardiomyocytes [ICMs]) has emerged as a cornerstone in our efforts to understand human cardiac development. This in vitro system effectively recapitulates essential hallmarks of cardiogenesis, including the activation of lineage-specific gene programs, the formation of sarcomeres, and the acquisition of functional electrophysiological profiles. Consequently, iCMs provide a physiologically relevant model for exploring fundamental developmental mechanisms, assessing drug-induced cardiotoxicity (Burnett et al. 2021), and modeling complex cardiac pathologies (Sala et al. 2019). Beyond that, iCMs represent a potential cell source for future regenerative therapies (Jiang and Lian 2020; Kadota et al. 2020). The in vitro differentiation of iPSCs to iCMs mirrors the sequential stages of embryonic heart formation, a complex process characterized by profound and highly orchestrated remodeling of the transcriptional landscape (Liu et al. 2017). Enhancing the physiological relevance of iCMs as an in vitro model requires a deeper understanding of the molecular processes governing cell fate specification (Bizy and Klos 2020). Although RNA sequencing has provided invaluable insights into these processes, analyses have primarily focused on differential gene expression (DGE), which quantifies the total change in a gene's transcriptional output. This approach, however, overlooks a crucial layer of regulatory complexity: the expression of distinct transcript isoforms. Alternative transcription and splicing generate multiple mRNA variants from a single gene, enabling the production of functionally diverse proteins that fine-tune cellular processes. During development, such isoform switching is essential for lineage commitment and functional maturation, yet its specific role in iCM differentiation remains insufficiently characterized. A major barrier to progress has been the inconsistent terminology used to describe isoform-level dynamics, which frequently conflates changes in transcript ratios with unproven functional outcomes at the protein level.

To address this gap, we propose a refined conceptual framework that distinguishes between three key modes of regulation: DGE, the change in the total transcriptional output of a gene; differential transcript expression (DTE), the change in the absolute abundance of an individual isoform; and differential transcript accumulation (DTA), the relative change in the proportional abundance of isoforms, independent of overall gene expression. Here, we use this framework for a comprehensive longitudinal transcriptomic study of iPSC-to-iCM differentiation. Our primary objective was to address two fundamental questions: first, the extent to which biological processes governed by DTA diverge from those regulated by conventional DGE and, second, whether stage-specific isoform transitions are mechanistically linked to the expression dynamics of RNA-binding proteins (RBPs). By dissecting these distinct regulatory layers, we aimed to construct a granular map of the cardiac developmental landscape and establish a robust methodology capable of dissecting regulatory mechanisms essential for cardiac maturation that are otherwise obscured in standard transcriptomic workflows.

## Results

### DTA is a prevalent feature of cardiac differentiation affecting specific transcript biotypes

**Figure 1.**
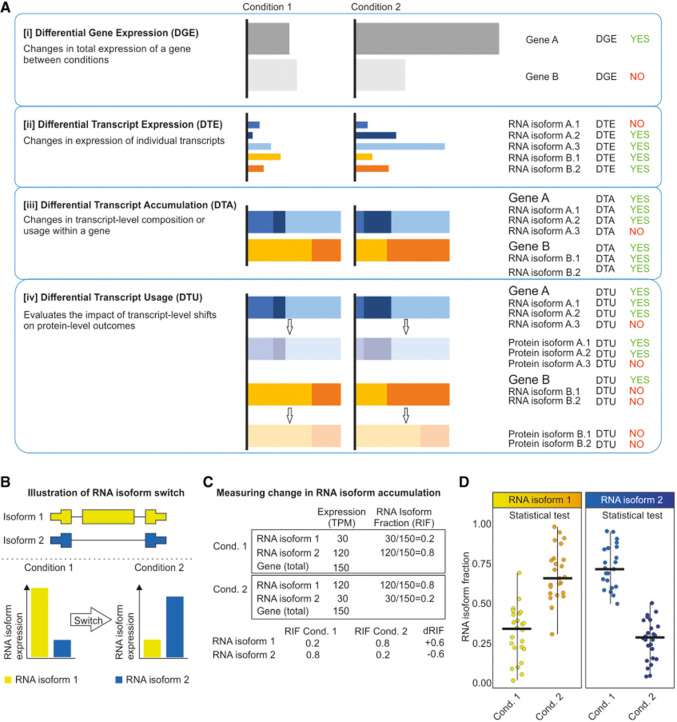
Conceptual framework for distinguishing gene- and isoform-level dynamics. (*A*) Comparative overview of differential analysis modes in transcriptomics. (*B*) Schematic representation of an RNA isoform switch. Illustration of a transcript-level isoform switch. (*Top*) Two RNA isoforms (yellow and blue) transcribed from a single gene locus with visible differences in their exon–intron architecture. (*Bottom*) Differential expression patterns of these isoforms across two biological states (condition 1 and condition 2). An isoform switch is characterized by a significant shift in the relative abundance of isoforms, in which the dominant transcript in the first condition (isoform 1) is replaced or superseded by an alternative variant (isoform 2) in the second condition. (*C*) Theoretical examples illustrating the calculation of RNA isoform fraction (RIF) and the difference in RIF (dRIF). (*Top*) In the tables, isoform-level expression values are provided in transcripts per million (TPM) for two conditions. RIF is determined by the ratio of a specific isoform's expression to the total gene expression (cumulative TPM of all isoforms). (*Bottom*) The dRIF metric quantifies the magnitude of the isoform switch, defined as the change in RIF values between states (*dRIF* = *RIF*_*Cond2*_ − *RIF*_*Cond1*_). (*D*) Statistical evaluation of RNA isoform fractions across biological replicates. Dot plots illustrate the distribution of RIF for individual isoforms across multiple samples (e.g., individuals) in the compared conditions. This framework allows for the statistical assessment of isoform-level variation by comparing RIF distributions between condition 1 and condition 2 for each specific transcript independently.

**Figure 2.**
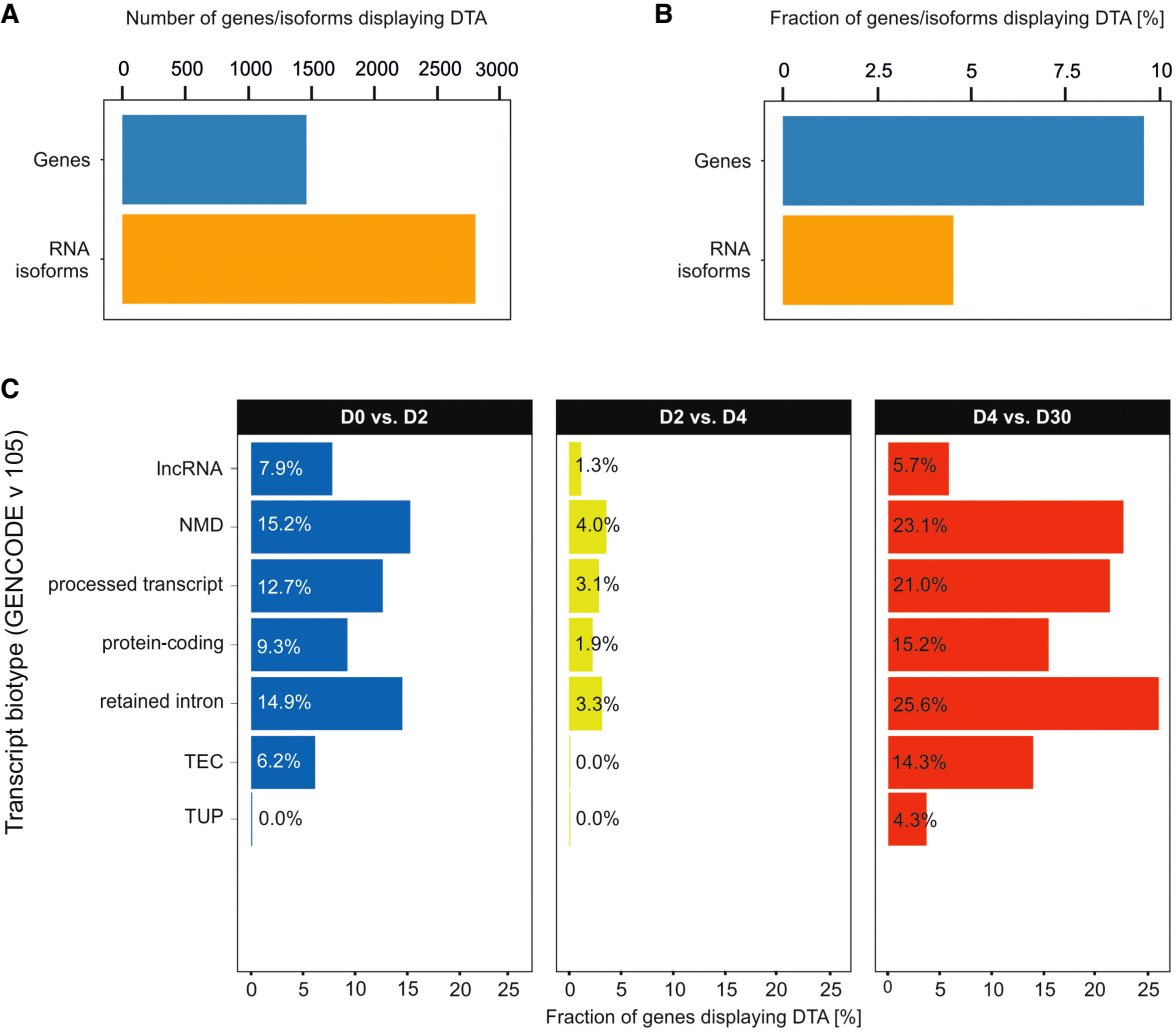
Quantification and biotype distribution of genes and isoforms undergoing DTA. (*A*) Total number of genes and RNA isoforms displaying DTA. (*B*) Fraction of genes and RNA isoforms displaying DTA identified across a iPSC-to-iCM differentiation time course. (*C*) Fraction of genes displaying DTA, grouped by transcript biotype (Ensembl release 115 taxonomy), during iPSC-to-iCM differentiation. (D0, D2, D4, D30) Days of differentiation, (lncRNA) long noncoding RNA, (NMD) nonsense mediated decay, (TEC) to be experimentally confirmed, and (TUP) transcribed unprocessed pseudogene.

To evaluate isoform dynamics throughout cardiomyogenesis, we used the proposed framework (Fig. 1A–D) to analyze three independent RNA-seq data sets, including two public ones (Liu et al. 2017; Bertero et al. 2019) and a novel data set covering four critical time points: D0 (pluripotency), D2 (cardiac mesoderm), D4 (cardiac progenitors), and D30 (early iCMs). Across the entire iPSC-to-iCM differentiation process, we detected 58,284 expressed transcript variants from 15,382 genes. Within this set, we identified 2851 high-confidence DTA-driven isoform switches occurring in 1484 unique genes (Fig. 2A; Supplemental Figs. S1, S2; Supplemental Table S1). This indicates that ∼10% (9.6%) of expressed genes undergo DTA, affecting 4.9% of all expressed isoforms (Fig. 2B).

We next examined the distribution of these DTA events across transcript biotypes (Fig. 2C). The “protein-coding” biotype contained the highest absolute number of isoform switches at each stage transition (258 in D0/D2, 36 in D2/D4, and 484 in D4/D30). A Fisher's exact test revealed that DTA was not randomly distributed; instead, it was significantly overrepresented in protein-coding, retained intron, and long noncoding RNA (lncRNA) transcripts (*P* < 0.01).

**Figure 3.**
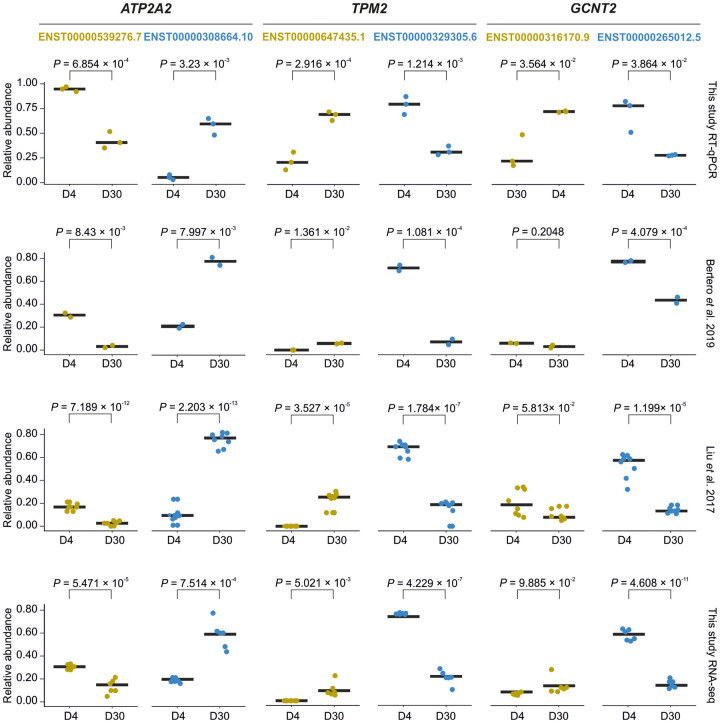
Replication and validation of DTA. Box plots showing the relative abundance of RNA isoforms evaluated with RT-qPCR (n = 3) and three RNA-seq data sets of iPSC-to-iCM differentiation: Bertero et al. (2019) (n = 2), Liu et al. (2017) (n = 8), and this work (n = 6). The black bars depict the mean value. Reference gene used: *ACTB*. Statistical significance was calculated with a Student's *t*-test for n > 5 and an unpaired two-tailed Welch's *t*-test for n < 5. (D4, D30) Days of iPSC-to-iCM differentiation.

To experimentally validate the computationally identified isoform switches, we used RT-qPCR to measure the relative abundance of specific transcript variants for three representative genes (*ATP2A2*, *TPM2*, and *GCNT2*) at D4 and D30. For *ATP2A2* and *TPM2*, the RT-qPCR results confirmed the direction of isoform shifts predicted from the RNA-seq data, with one major isoform increasing in abundance and the other decreasing at D30 relative to D4 (Fig. 3). Although the primary isoform switch for *GCNT2* was also validated, a secondary isoform showed a discrepancy between the methods. Overall, these results confirm that our analysis pipeline accurately identifies DTA-driven changes in isoform ratios.

### DTA and DGE represent distinct regulatory programs with unique temporal dynamics

**Figure 4.**
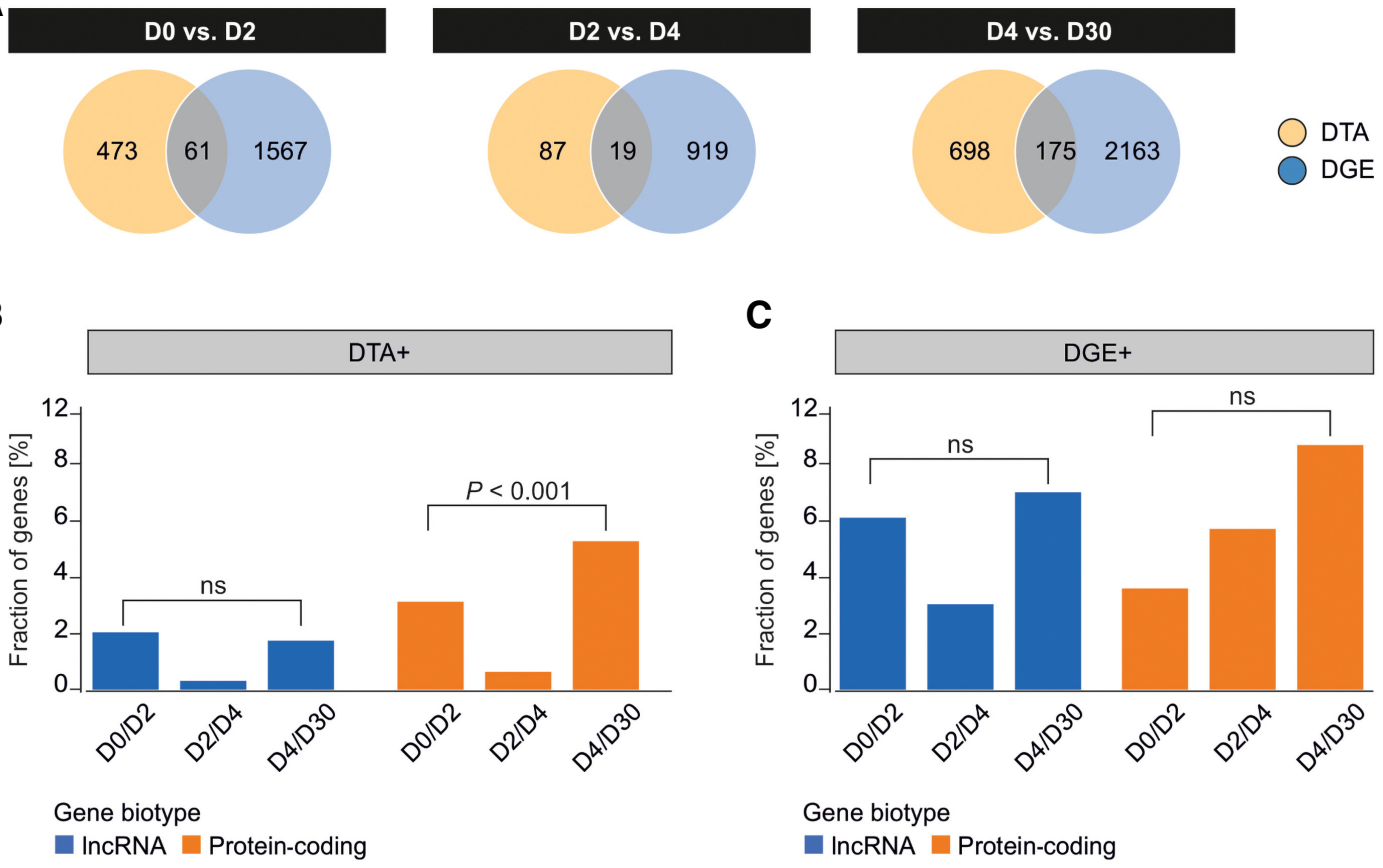
Distribution of DGE and DTA. (*A*) Number of genes displaying DGE (blue) or DTA (yellow) during the iPSC-to-iCM differentiation. The overlapping region shows several genes displaying both DGE and DTA, Percentage of lncRNA (blue) and mRNA coding (orange) genes displaying DGE (*B*) or DTA (*C*) during the iPSC-to-iCM differentiation. Statistical significance was calculated with a Student's *t*-test (n = 16). (D0, D2, D4, D30) Days of differentiation.

To determine the relationship between isoform-level and gene-level regulation, we compared the sets of genes exhibiting DTA and DGE at each stage transition. At all intervals, more genes were regulated by DGE than by DTA: 534 versus 1628 in D0/D2, 106 versus 938 in D2/D4, and 873 versus 2338 in D4/D30 (Fig. 4A; Supplemental Tables S1, S2).

Crucially, the overlap between these two regulatory modes was minimal. The fraction of genes displaying both DTA and DGE was only 11% in D0/D2, 18% in D2/D4, and 20% in D4/D30 (Fig. 4A). To confirm that the observed DTA events reflected true changes in isoform abundance and were not artifacts of overall gene expression shifts, we correlated DTA with DTE and DGE. We found that DTA was strongly correlated with DTE (Pearson *r* > 0.6, *P* < 0.05) but showed no correlation with DGE (Pearson *r* < 0.1, *P* > 0.5), confirming their independence.

This regulatory distinction is further highlighted by divergent temporal dynamics during differentiation, particularly among protein-coding genes (Fig. 4B,C). The number of protein-coding genes exhibiting DTA increased significantly from the early (D0/D2) to the late (D4/D30) stage of differentiation (Mantel–Haenszel test, *P* < 0.001) (Fig. 4B). In contrast, the number of protein-coding genes regulated by DGE did not change significantly over the same period (*P* > 0.05) (Fig. 4C). This contrast suggests an increasingly specialized role for isoform switching during cardiomyocyte maturation.

### Alternative transcription drives isoform switches that alter protein-coding potential and function

**Figure 5.**
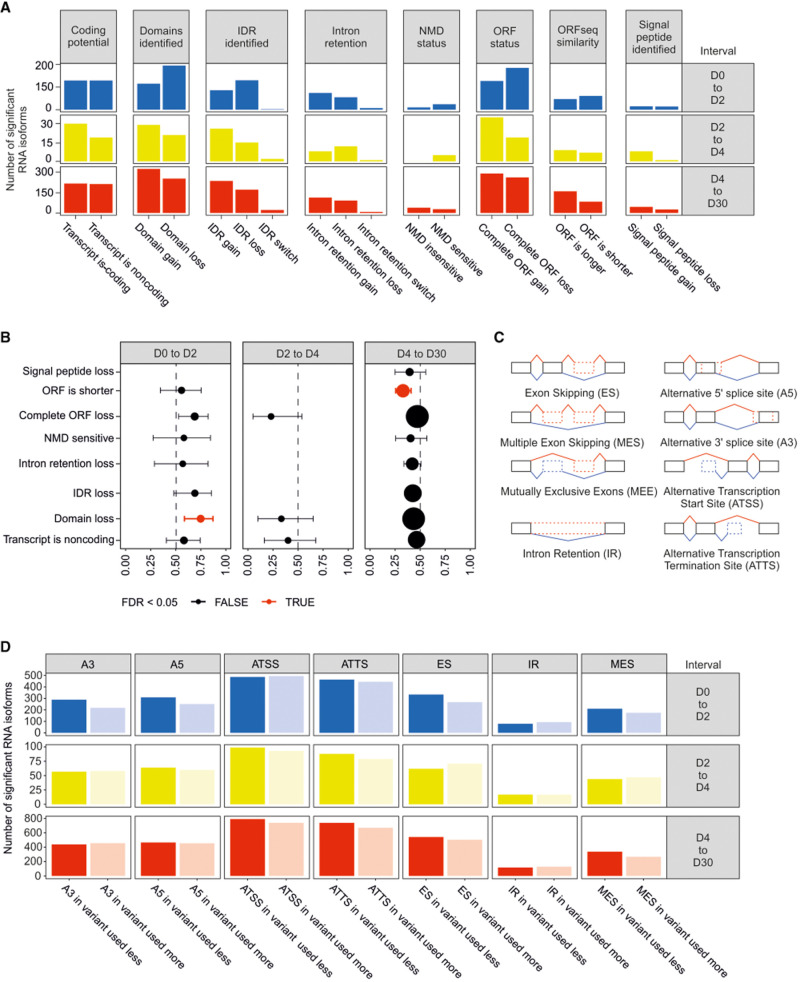
Alternative splicing mechanisms associated with RNA isoform switches and consequences for protein accumulation. (*A*) Number of events associated with particular functional implications to the RNA isoform. (*B*) Statistical significance of events related to selected functional consequences to the RNA isoform. (*C*) Illustration of alternative transcription and splicing events. (*D*) Number of events with identified alternative transcription or splicing events during the iPSC-to-iCM differentiation. (A3) Alternative 3′ splice site, (A5) alternative 5′ splice site, (ATSS) alternative transcription start site, (ATTS) alternative transcription termination site, (ES) exon skipping, (IR) intron retention, (MES) multiple exon skipping, and (D0, D2, D4, D30) days of differentiation.

We first analyzed the potential functional impact of the identified isoform switches on the resulting transcripts. DTA events were most frequently associated with the gain or loss of protein domains, open reading frame (ORF) changes, and shifts in coding potential (Fig. 5A). These functional consequences were stage specific: The exit from pluripotency (D0–D2) was significantly enriched for isoforms characterized by protein domain loss, whereas the final maturation stage (D4–D30) was marked by the preferential selection of isoforms with significantly longer ORFs (FDR < 0.05) (Fig. 5B; Supplemental Fig. S3).

To further investigate the impact of altered coding potential, we analyzed genes undergoing DTA-driven switches between noncoding and protein-coding dominant isoforms during maturation (D4–D30). Genes transitioning toward protein-coding isoforms were significantly enriched for pathways associated with muscle contraction and actin filament movement (Supplemental Fig. S4; Supplemental Table S3). Notably, this enrichment profile mirrored that of the entire DTA-regulated gene set. In contrast, genes exhibiting the opposite switch, characterized by the downregulation of coding isoforms relative to noncoding ones, were specifically enriched for RNA processing pathways and showed a depletion of muscle-related GO terms.

To identify the molecular mechanisms driving these structural changes, we quantified the relative contribution of different alternative splicing and transcription events. Across all developmental stage-to-stage transitions, alternative transcription events—specifically alternative transcription start sites (ATSSs) and alternative transcription termination sites (ATTSs)—were consistently more prevalent than any type of alternative splicing event (Fig. 5C,D). These results identify alternative transcription as the predominant driver of DTA throughout the cardiomyocyte differentiation trajectory in vitro (Fig. 5D).

Furthermore, we investigated whether isoforms characterized by specific molecular features, namely, those generated via particular splicing events, exhibited preferential accumulation. However, we found no significant association between these features and differential isoform accumulation.

To evaluate the biological relevance of isoform-level regulation, we examined DTA events in representative canonical cardiac and noncardiac genes. Several key cardiac regulators, including *TTN*, *TNNT2*, *TNNI3*, and *TPM1*, exhibited clear isoform shifts between D4 and D30 (Supplemental Table S1; Supplemental Fig. S5). The transitions were consistent with known fetal-to-mature isoform switches required to optimize sarcomere architecture and contractile function. Beyond these expected changes, we identified significant DTA in genes not classically associated with cardiac-specific functions, such as *QKI*, *RBFOX2*, *PKM*, and *VEGFA*. These results indicate that transcriptomic remodeling during cardiomyocyte differentiation extends beyond structural and contractile components to include regulatory, metabolic, and signaling pathways, potentially driving further cardiomyocyte maturation and specialization.

### Genes regulated by DTA and DGE are enriched in distinct biological pathways

**Figure 6.**
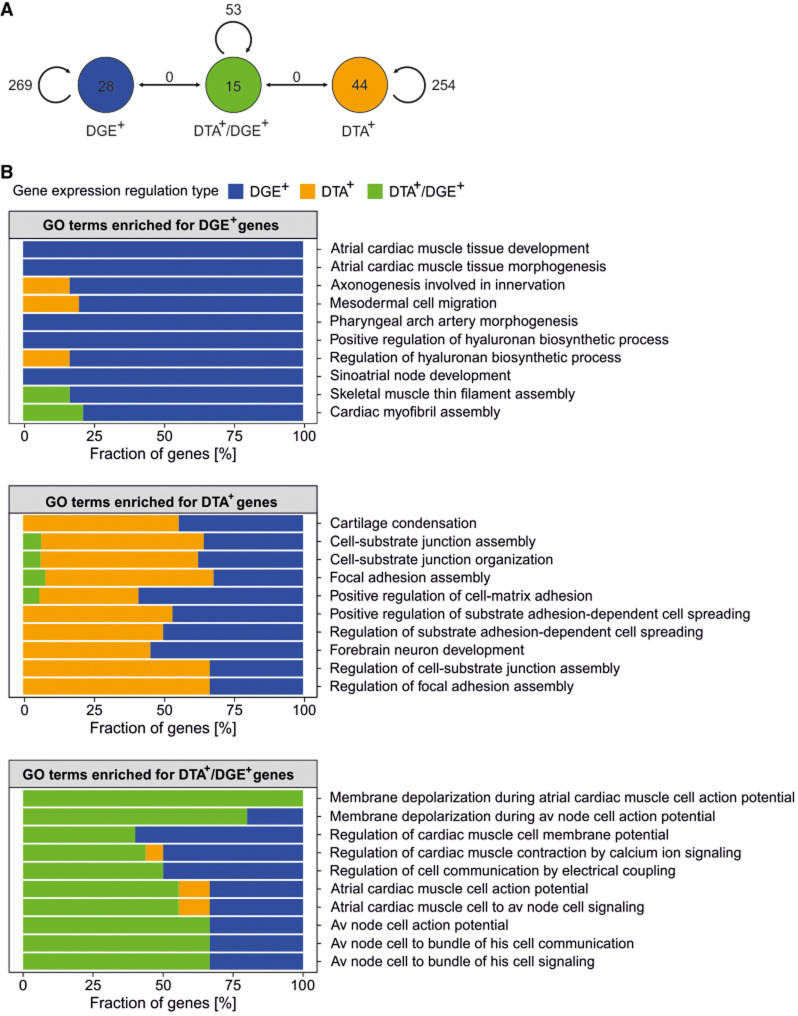
Functional annotation of genes displaying DGE and DTA. (*A*) GO biological process terms enriched for genes displaying DTA, DGE, or DTA/DGE. Circles denote GO terms; edges denote the number of shared edges between GO terms, when the similarity score was >0.3 (Supplemental Fig. S8). (*B*) Barplots depicting percentage composition of the top 10 GO terms enriched in gene sets displaying DTA, DGE, or DTA/DGE. Colors denote which type of gene expression regulation displayed genes which formed each GO term.

To determine if DTA and DGE regulate distinct cellular functions, we performed a pathway enrichment analysis on genes identified during the D4 to D30 maturation stage. Genes were categorized into three groups (Fig. 6A) based on their regulatory mode: those coregulated by both DTA and DGE (DGE^+^/DTA^+^), those regulated exclusively by DTA (DGE^–^/DTA^+^), or by DGE (DGE^+^/DTA^–^).

Gene Ontology (GO) analysis demonstrated that each regulatory group governs functionally distinct and largely nonoverlapping biological processes (Fig. 6B; Supplemental Figs. S8, S9; Supplemental Table S4). Genes subjected to dual regulation (DGE^+^/DTA^+^) were enriched for fundamental cardiomyocyte-specific pathways, including cardiac conduction and calcium signaling. In contrast, genes regulated exclusively by DTA were associated with processes such as RNA splicing and cell junction assembly. Finally, the gene set controlled only by DGE was primarily enriched for developmental morphogenesis pathways. These findings suggest that DTA and DGE represent separate regulatory layers that target distinct functional modules during cardiomyocyte maturation.

### DTA-regulated pathways are specific to contractile cardiomyocytes

**Figure 7.**
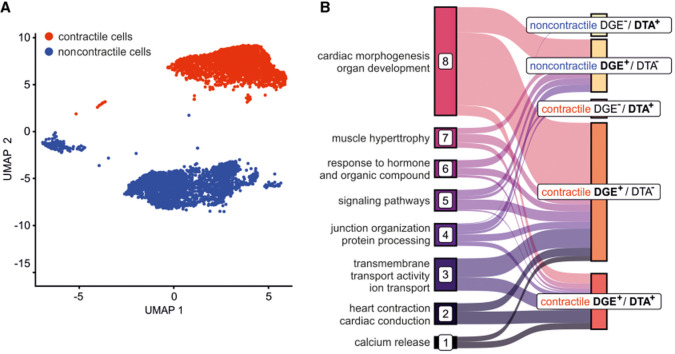
Cell type specificity of biological processes enriched in DGE and DTA displaying genes. (*A*) UMAP embedding of D30 with contractile iCM (red) and noncontractile (blue) cells. (*B*) The Sankey plot of the enriched GO biological process pathway clusters and their relation to cell–gene regulation-type-specific groups.

To assign bulk gene regulatory signatures at cell type–specific resolution within the heterogeneous D30 culture, we analyzed a single-cell RNA-seq data set to distinguish contractile (iCM) from noncontractile populations (Fig. 7A). We then mapped the biological pathways associated with the three regulatory groups (DGE^+^/DTA^+^, DGE^−^/DTA^+^, and DGE^+^/DTA^–^) to these distinct cell types.

The analysis revealed a divergence in cell type specificity. Pathways associated with any form of DTA (DGE^+^/DTA^+^ and DGE^−^/DTA^+^) were almost exclusively enriched in the contractile iCM population. These iCM-specific pathways encompassed core cardiac processes, including cardiac muscle development, atrioventricular (AV) node signaling, ion signaling, and cell junction organization (Fig. 7B; Supplemental Figs. S6, S7, S10; Supplemental Table S5). In stark contrast, pathways controlled solely by DGE (DGE^+^/DTA^–^) showed broad enrichment across both contractile and noncontractile cells.

This regulatory split was evident even within shared biological processes. For instance, RAS protein signal transduction was controlled by DTA-displaying genes in contractile cells, whereas in the noncontractile population, genes within this same pathway were regulated by DGE. These findings establish DTA as a hallmark of functional specialization during cardiomyocyte maturation, whereas DGE represents a more generalized mode of gene regulation across diverse cell types.

### Stage-specific expression of RBPs correlates with isoform switching events

**Figure 8.**
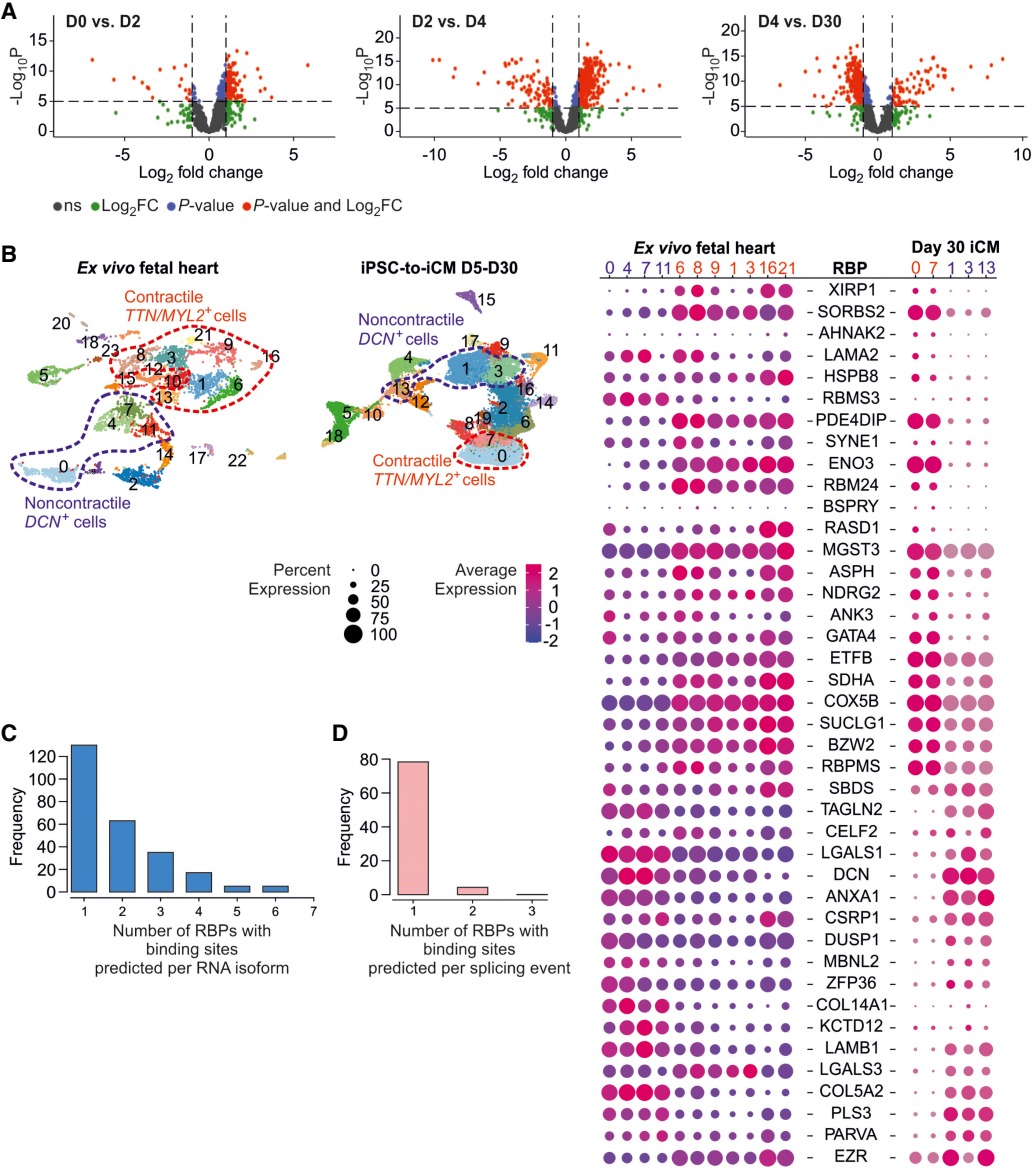
Analysis of RBP expression dynamics in cardiac cells. (*A*) Volcano plots showing differential RBP gene expression across sequential time point comparisons during iPSC-to-iCM differentiation. (D0, D2, D4, D30) Days of differentiation. (*B*) Dot plot representing single-cell RBP gene expression for candidates upregulated in D30 iCMs across contractile and noncontractile cells in ex vivo fetal heart tissue and D30 in vitro iCMs. (*C*) Histogram of DeepBIND-predicted RBP binding sites frequencies in transcripts displaying DTA. (*D*) Histogram of DeepBIND-predicted RBP binding sites frequencies in sequences flanking DTA-associated splice sites.

To identify potential upstream regulators of DTA, we analyzed the expression of 1731 known RBPs throughout the differentiation time course (Supplemental Table S6). Among these, 438 RBPs exhibited significant differential expression. The overall trend shifted from a net upregulation of RBPs during early stages (D0–D4) to a net downregulation during cardiomyocyte maturation (D4–D30) (Fig. 8A). From the cardiac progenitor stage (D4) onward, we observed consistent expression trajectories, with 53 RBP genes being continuously upregulated and 137 being continuously downregulated through D30. This included the sustained upregulation of known cardiac splicing regulators, such as *XIRP1* and *SORBS2*, and the progressive downregulation of pluripotency-associated RBP genes, including *TRIM71* (Supplemental Table S7).

We next examined the cell type specificity of these RBPs at D30 using single-cell RNA-seq data from both iCM cultures and fetal human heart tissue. The majority of the RBPs upregulated during the D4 to D30 transition were differentially expressed between contractile and noncontractile cells. Transcripts for key cardiomyocyte differentiation factors, including the transcription factor GATA4 and the splicing regulator RBM24, were significantly enriched in contractile cells. Conversely, mRNAs of RBPs like DCN and ANXA1 were enriched in the noncontractile population (Fig. 8B).

Finally, to mechanistically link these RBPs to the observed isoform switches, we performed computational motif enrichment analysis within the sequences flanking the D4 to D30 alternative splicing events sites. This analysis revealed significant enrichment for predicted binding sites of a small set of differentially expressed RBPs, specifically U2AF2, KHDRBS1, CPEB4, and RBM24 (Supplemental Fig. S11; Supplemental Tables S8, S9). Although individual transcripts often harbored combinations of these binding sites (Fig. 8C), analysis of individual splice junctions most frequently identified motifs for a single RBP, with U2AF2 being the most predominant (Fig. 8D). These findings suggest that a core set of dynamically expressed, cell type–specific RBPs serves as the primary regulators of the alternative splicing events shaping cardiomyocyte maturation.

To complement our DeepBind-based RBP analysis, we cross-referenced our DTA events with published high-confidence target lists for RBM20 and QKI, two cardiac splicing regulators for which trained DeepBind models are not available (Supplemental Table S11). For RBM20, we used the HITS-CLIP target list from Maatz et al. (2014), which provides experimental binding evidence ranked by the number of reproducible clusters (hits_total). Of 733 RBM20 HITS-CLIP targets, 81 (11.1%) showed DTA events during iPSC-to-CM differentiation. This proportion increased with binding confidence: Among the 11 highest-confidence targets (hits_total ≥ 10), six (54.5%) showed DTA, and among the top five targets (hits_total ≥ 20), four (80%) showed DTA. The top RBM20 targets with DTA included *TTN*, *PDLIM5*, *SLC8A1*, and *SORBS2*, with isoform switching predominantly occurring at the D4/D30 transition (Supplemental Table S12). Notably, RBM20 itself showed DTA at the D2/D4 transition, suggesting autoregulatory control of its own splicing during differentiation. For QKI, cross-referencing with experimentally validated targets from Chen et al. (2021) and Montañés-Agudo et al. (2023) revealed that seven published QKI targets showing DTA events included *TTN*, *NEBL*, *ABLIM1*, *PDLIM5*, *TPM1*, *CAMK2D*, and *ACTN2*. DTA events for QKI targets were again predominantly observed at the D4/D30 transition, with the exception of *NEBL*, which switched at D0/D2 (Supplemental Table S13). Importantly, *TTN*, *PDLIM5*, and *TPM1* appeared as DTA-positive targets of both RBM20 and QKI, pointing to potential coregulation of key sarcomeric transcripts by multiple RBPs.

## Discussion

Vast transcriptomic changes take place during cell differentiation. Until now, the regulation of gene expression during iPSC-to-iCM differentiation has been studied mainly at the level of individual genes, overlooking the changes at the level of RNA isoforms. Here, we investigated the DTA across several cell culture protocols to better our knowledge of the mechanisms regulating gene expression during iPSC-to-iCM differentiation.

We demonstrated that many regulators of the iPSC-to-iCM differentiation are subject to DTA. DTA was also associated with many novel genes involved in the iPSC-to-iCM differentiation process, which standard DGE analysis misses. The DTA-displaying genes were involved in biological processes distinct from those involving genes regulated solely through DGE and included cell junction development and RNA processing. We observed that a subset of genes displayed a relative isoform accumulation shift from noncoding to coding isoforms during the differentiation. DTA was significantly associated with particular transcript and gene biotypes. Pathways enriched in genes displaying DTA were cell type specific between contractile and noncontractile cells. Further, we established that the expression of genes encoding RBPs, potentially involved in the generation of particular RNA isoforms, became consistently more or less pronounced, indicating a directional trend of expression already at the cardiac mesoderm stage, therefore having a substantial impact on cell fate very early in the iPSC-to-iCM differentiation trajectory as well as in early maturation stage of iCMs.

We observed that ∼10% of genes expressed during the iPSC-to-iCM differentiation displayed DTA across multiple data sets, showing that this mechanism is a common and recurrent process during the acquisition of cardiac fate by the differentiating cells. The extensive usage of DTA for gene expression control was previously described regarding cancer and infection response (Vitting-Seerup and Sandelin 2017; Chang et al. 2022; Kakuk et al. 2023). Furthermore, we have concluded that DTA and DGE primarily operate on distinct sets of genes. It remains to be understood why some genes display DTA whereas others display DGE, as well as why the cell produces more of the same RNA isoform for some of the genes whereas multiple different RNA isoforms are generated for others. Our results indicate some functional patterns when using both mechanisms. The genes displaying solely DTA were coding for proteins with functions that might demand faster response time, that is, engaged in cell junction organization, RNA splicing, processing protein localization, or response to metabolic changes. At the same time, the genes displaying DGE were mainly coding for proteins that have critical structural functions in cardiac tissue, namely, engaged in muscle cell differentiation and cardiac tissue morphogenesis. Between them were placed protein products encoded through DTA and DGE mechanisms responsible for both critical cardiac functions and demanding fast response time, including cardiac conduction, calcium signaling, and cell action potential. These results suggest that the utilization of DTA or DGE mechanisms is linked with the function of the protein isoform or noncoding RNA. Such compensatory changes across transcripts balancing out the overall gene expression were previously reported in Parkinson's disease (Dick et al. 2020). However, no in-depth analysis of these gene sets was performed.

DTA or DGE display was associated with the gene biotype throughout the iPSC-to-iCM differentiation. This phenomenon played an increasingly important role in regulating the expression of protein-coding genes during subsequent transitions until the early mature iCM stage. This association was not observed for the genes displaying DGE. This suggests that DTA is important in fine-tuning gene expression in the late stages of iPSC-to-iCM differentiation. This finding aligns well with general biological phenomena; whereas cells progress toward a more specialized state, the regulation of gene expression becomes increasingly sophisticated. DTA allows for the utilization of multiple transcript variants from a single gene, enabling precise regulation of gene expression and transcription products diversity. This is particularly critical in the final stages of differentiation, in which specific protein isoforms are required to fulfill the specialized functions of CMs. The heightened prevalence of DTA in the final stages of iPSC-to-iCM differentiation further underscores its importance in their maturation and functionality. This suggests that transcript isoform diversity is a key regulatory mechanism in the terminal differentiation phase, likely contributing to the establishment of the mature CMs phenotype. Furthermore, the absence of a similar increase in DGE highlights a fundamental distinction between the regulatory roles of DTA and DGE. Although DGE provides a broad regulation of gene expression levels, DTA offers a more nuanced control, ensuring that the specific isoforms necessary for CM function are expressed at the appropriate stages in the appropriate amount relative to the total gene output.

The observation that both cardiac and noncardiac genes display transcript-level remodeling underscores the widespread regulatory impact of alternative transcript accumulation during cardiomyocyte differentiation. Isoform transitions in canonical cardiac genes such as *TTN*, *TNNT2*, and *TPM1* are consistent with known developmental splicing programs that tune contractile and structural properties of the maturing heart. However, the detection of DTA in genes not traditionally linked to cardiac biology, including *QKI*, *RBFOX2*, *PKM*, and *VEGFA*, suggests that isoform regulation also influences broader regulatory and metabolic pathways. These noncanonical changes may reflect adaptive remodeling of RNA processing, signaling, and energy metabolism necessary for establishing a mature cardiomyocyte phenotype. Together, these findings highlight the potential of isoform-level analyses to uncover previously unrecognized layers of regulation that complement conventional gene-level perspectives in cardiac development.

The observed noncoding to coding isoform switch in early iCMs raises intriguing questions about the role of transcriptional priming in differentiation. Although genes exhibiting DTA-associated coding potential gain in upregulated isoforms were enriched for muscle contraction pathways, a finding seemingly aligned with priming, this enrichment mirrored patterns in all DTA genes regardless of the increase or decrease in the isoform accumulation. This suggests the signal may reflect the overall enrichment of these pathways in DTA-displaying genes rather than directed priming. Conversely, downregulated coding isoforms were linked to RNA processing pathways, further complicating the priming hypothesis. Despite these challenges, the disproportionate association of coding potential switches with cardiac conduction and RNA-binding genes implies that DTA may serve dual roles: facilitating functional maturation of CMs while maintaining regulatory flexibility through isoform dynamics. These findings underscore the complexity of isoform regulation in lineage commitment, in which transcriptional priming could coexist with broader transcriptomic remodeling tied to cell identity.

Our findings revealed a divergence in regulatory logic between contractile and noncontractile cell populations during iPSC-to-iCM differentiation. Pathways enriched in genes displaying DTA were specific to contractile CMs, enriching for core functional modules such as cardiac muscle development, ion channel signaling, and sarcomere organization. Notably, these processes, critical for electromechanical coupling and force generation, relied on isoform switching (DTA) rather than bulk expression changes (DGE). For example, calcium-mediated signaling, essential for CM contraction, was modulated in contractile cells through both DTA and DGE mechanisms, suggesting dual layers of regulation to calibrate excitation–contraction dynamics. Conversely, pathways active in noncontractile cells, such as RAS protein signal transduction, exhibited distinct regulatory strategies: Although contractile cells employed DTA to fine-tune RAS-related genes, noncontractile cells relied on DGE to upregulate these pathways. This dichotomy underscores that DTA is a hallmark of functional specialization in maturing cardiomyocytes, enabling precise control of cardiac-specific processes without altering overall gene expression. These results redefine transcriptional regulation during differentiation: Isoform switching is not merely a secondary layer of control but a central mechanism for establishing cell identity. By decoupling transcript diversity from expression magnitude, DTA allows contractile cells to optimize functional maturation (e.g., sarcomere assembly) while maintaining transcriptional flexibility. This paradigm shift highlights the need to integrate isoform-resolved analyses into studies of cellular commitment, particularly in systems requiring high functional precision, such as cardiomyocytes.

The temporal dynamics of RBP expression further underscore the critical role of DTA in guiding cell fate determination during iPSC-to-iCM differentiation. We observed that many DE RBP genes exhibit sustained up- or downregulation beginning at early differentiation stages, either during the exit from pluripotency or at the cardiac mesoderm stage, and maintain these expression trends through iCM maturation. Notably, many of these RBPs become specific to contractile CMs, and their predicted target transcripts overlap significantly with genes displaying DTA, particularly those involved in cardiac-specific processes such as ion signaling and sarcomere organization.

This coordinated regulation suggests that RBPs act as early orchestrators of lineage commitment, shaping the transcript isoform landscape to restrict differentiation toward a CM fate. For example, RBPs upregulated during cardiac mesoderm specification (e.g., XIRP1, SORBS2) likely drive isoform switching in genes critical for contractile function, whereas downregulated RBPs (e.g., TRIM71, PSIP1) may dismantle pluripotency-associated splicing programs. Together, these findings position RBPs as key regulators of DTA-mediated cell fate restriction, operating from the earliest stages of differentiation to ensure precise transcriptional and functional maturation of iCMs. Our cross-referencing with published RBM20 and QKI target lists supports a role for these splicing regulators in shaping isoform switching during cardiomyocyte differentiation. The increasing proportion of DTA events with RBM20 binding confidence (80% among top targets) suggests that stronger binding is associated with functional regulation. The overlap of RBM20 and QKI targets, including *TTN*, *PDLIM5*, and *TPM1*, points to potential coregulation of key sarcomeric genes. The predominance of DTA events at the D4/D30 transition indicates that both RBPs primarily contribute to late-stage splicing changes. Additionally, the observed autoregulation of RBM20 is consistent with known feedback mechanisms in splicing factor biology. As this analysis relies on published target data sets derived from rodent models, further human-specific binding and functional studies will be needed to confirm these regulatory roles.

To conclude, this work's main contribution was to show that DTA plays an important role in fine-tuning gene expression and cardiomyocyte-specific functions during iPSC-to-iCM differentiation. We suggest that integrating DTA analysis with traditional gene expression studies is crucial for a comprehensive understanding of cardiac development and function.

### Limitations of the study

Although this study provides a comprehensive analysis of isoform dynamics in cardiomyocyte differentiation, we acknowledge several limitations that offer avenues for future investigation.

First, our study is constrained by the design of the in vitro time course. We observed a marked reduction in DTA and DGE events during the highly dynamic D2/D4 transition, which may reflect a combination of technical challenges in achieving precise cell synchronization across replicates and a potential biological bottleneck in transcriptional activity. Furthermore, our study is limited by the large temporal gap between the cardiac progenitor stage (D4) and early cardiomyocytes (D30). This design prevents a granular assessment of the dynamics of isoform switching during the critical phase of terminal differentiation and sarcomere assembly, for which intermediate time points would be required.

Second, our findings are based on short-read RNA-seq, which has inherent constraints. Our conclusion that alternative transcription is the dominant mechanism driving DTA should be interpreted with caution, as the accurate assembly and quantification of full-length, complex isoforms from short reads can be challenging. Definitive validation of these findings will require long-read sequencing technologies (e.g., Pacific Biosciences [PacBio] or Oxford Nanopore Technologies [ONT]). Similarly, our mapping of bulk signatures to single-cell populations relies on marker-based classification, which can be affected by technical artifacts such as gene dropout.

Further research should also investigate if the identified isoform switches result in functional changes at the protein level or alter cellular physiology. This distinction is crucial, as our DTA framework describes changes at the RNA level; confirming true “differential transcript usage” (DTU) would require proteomic evidence that these isoform shifts result in a corresponding diversity of protein isoforms. Future studies are therefore needed to validate these predictions using proteomics and to assess their impact on cardiomyocyte properties such as contractility, calcium handling, and electrophysiology.

The predicted interactions between RBPs and their target transcripts are also inferential and require mechanistic experiments, such as CLIP-seq to map in vivo RBP binding sites, followed by functional studies using tools like antisense oligonucleotides to modulate specific isoforms and observe the cellular response.

Similarly, essential conclusions on the compensatory mechanism of gene expression regulation could be drawn from experiments utilizing antisense oligonucleotides for targeted activation or repression of specific RNA isoforms (Bartyś et al. 2022).

## Methods

### Identification of DTA and DGE

To comprehensively map RNA isoform dynamics during cardiac differentiation, we analyzed three independent RNA-seq data sets—two previously published by Liu et al. (2017), and Bertero et al. (2019), as well as one newly generated at four key time points: D0 (iPSC), D2 (cardiac mesoderm), D4 (cardiac progenitors), and D30 (early iCMs). For bulk RNA-seq data sets, raw paired-end FASTQ files were preprocessed with Trim Galore! (parameters: ‐‐quality 20, ‐‐illumina, ‐‐length 20, ‐‐trim-n; https://github.com/FelixKrueger/TrimGalore). The filtered reads were then quasi-aligned to the GENCODE v39 reference human transcriptome using Salmon v1.6.0 (parameters: -l A ‐‐validateMappings ‐‐numGibbsSamples 20).

The resulting transcript abundance and count tables were loaded into R v4.1 (R Core Team 2025) with importIsoformExpression for DTA analysis by the IsoformSwitchAnalyzeR package (Vitting-Seerup and Sandelin 2019) and with import_counts for the analysis with the DTUrtle package (Tekath and Dugas 2021). Both packages were run with default settings. In IsoformSwitchAnalyzeR, the applied method to identify DTA was DEXSeq: FDR cutoff 0.05, dRIF cutoff 0.1, features must contribute at least 5% of the total expression, and gene expression cutoff at one transcript per million (TPM). In DTUrtle, the method applied for identifying DTA was DRIMSeq: filtering_strategy = bulk. Features must contribute at least 5% of the total expression in at least 50% of the samples of the smallest group, and the total gene expression must be five TPM or more for at least 50% of the samples of the smallest group. To ensure robustness, only events detected by both tools and present in at least two independent data sets were retained for downstream analysis.

DGE was performed from the DTUrtle by applying the DESeq2 method (Love et al. 2014). Only those DGE events identified in at least two independent data sets were used in downstream processing. The overlap of DTA and DGE events identified across all three data sets was highly significant (phyper *P* < 1 × 10^−15^), confirming the robustness of our findings.

### Functional annotation of splicing events

To perform functional annotation of the isoform switching events, we extracted the coding sequences of the transcripts and performed in silico prediction of the transcript's coding potential (software CPAT) (Wang et al. 2013), protein domains (software Pfam) (Mistry et al. 2021), signal peptides (software SignalP) (Teufel et al. 2022), and intrinsically disordered domains (software IUPred2A) (Mészáros et al. 2018).

### Single-cell RNA-seq data processing

Raw sequencing data were processed with Cell Ranger count (v3.0.2). Cell Ranger output was then processed in Seurat v4.2 (Hao et al. 2024). Cells were excluded from the analysis unless they met the following criteria: nFeature_RNA > 500, nCount_RNA > 2000, percent mitochondrial reads <25, percent ribosomal reads <25. SCTransform was used to perform normalization, variance stabilization, and feature selection. Thirty principal components were used for UMAP embedding in two dimensions. Clustering was done at resolution 0.5. Differential expression of the genes was done between cell clusters identified as contractile (expression of *TTN* and *MYH6* or *MYH7*) and noncontractile (expression of DCN and lack of expression for *TTN*, *MYH6*, and *MYH7*), as significantly DE genes were considered the ones with FDR < 0.01.

### Cell culture and CM differentiation

In this study, three different human iPSC lines were used: two male lines from the hPSCreg repository, WTSIi322-A (RRID:CVCL_EE98) and WTSIi250-B (RRID:CVCL_AJ83), and a female cell line obtained from GMO7522 fibroblasts. The pluripotency state of the obtained cells was characterized by SOX2 and NANOG immunochemistry. The iPSCs were cultured on Geltrex (Thermo Fisher Scientific A1413301)-coated 12-well plates in Essential 8 medium (Thermo Fisher Scientific A1517001). iPSCs were next differentiated to iCMs using a PSC cardiomyocyte differentiation kit according to the manufacturer's instructions (Thermo Fisher Scientific A2921201). After 8 days, cells became contracting iCMs and were further cultured in PSC cardiomyocyte maintenance medium (Thermo Fisher Scientific A2920801) for at least 30 days. To further increase cardiomyocyte purity, the differentiated cells were subjected to subsequent glucose starvation using non-glucose-supplemented RPMI/B27 medium to decrease noncardiomyocyte cells. TNNT2 expression was used as a measure of differentiation efficiency. All cells in culture were maintained at 37°C in incubators with 5% CO_2_.

### RNA extraction and cDNA synthesis

The RNA was extracted from the iCMs on day 4 and day 30. Cells were first washed with PBS and dissociated from the plate either by 3 min incubation in 37°C with PBS with EDTA (4 day iCMs) or 5–10 min incubation with TrypLE (Thermo Fisher Scientific 12604013; 30 day iCMs). Dissociated cells were washed with medium from the plate and collected in 15 mL tubes. Centrifugation for 3 min at 300*g* at RT yielded a cell pellet that was washed with cold PBS twice and centrifuged for 4 min at 600*g* and 4°C. Finally, collected cells were resuspended in 1 mL of TRIzol reagent (Thermo Fisher Scientific 15596026) or 200 µL of RNAlater (Sigma-Aldrich R-0901) and stored at –20°C for further RNA extraction. The standard phenol/chloroform protocol was used to isolate RNA from TRIzol reagent samples. Total RNA isolation from RNAlater-collected samples was conducted with mirVana miRNA isolation kit (Thermo Fisher Scientific AM1561). The amount and quality of the obtained RNA were measured on a Multiskan SkyHigh microplate spectrophotometer (Thermo Fisher Scientific). The 500 ng of RNA was used as a template to generate complementary DNA (cDNA) with a SuperScript IV first-strand synthesis system with ezDNase enzyme (Thermo Fisher Scientific 18091150). Reverse transcription was conducted using a thermal cycling program: 10 min at 23°C, 10 min at 55°C, and 10 min at 80°C on the T100 thermal cycler (Bio-Rad). The obtained cDNA was diluted in RNase-free water to a final concentration of 10 ng/µL for qPCR and stored at –20°C.

### RNA-seq

For each sample, RNA libraries were prepared from 100 ng of RNA using the KAPA RNA hyperprep kit with RiboErase (Kapa Biosystems) according to the manufacturer's protocol, followed by a quality check using a fragment analyzer (Agilent). The libraries were normalized, pooled, and sequenced using the NovaSeq 6000 platform (Illumina), yielding an average of 120 million 151 bp paired-end reads per sample.

### Primer design and quantitative PCR analysis

For each target gene, three pairs of primers were designed: (1) one pair to amplify a constitutive region common to both isoforms and (2) two isoform-specific pairs to distinguish between individual transcripts. Isoform structures were visualized, and unique sequences were identified using the Integrated Genome Browser (v9.1.8) (Freese et al. 2016). Primers were designed using Primer3 (Untergasser et al. 2012) and validated with the IDT OligoAnalyzer tool. Sequences are provided in Supplemental Table S10. All oligonucleotides were synthesized by Thermo Fisher Scientific.

qPCR reactions were prepared using PowerTrack SYBR green master mix (Thermo Fisher Scientific A46110), 10 ng of cDNA, and a 10 µM mix of forward and reverse primers. Amplification was conducted in triplicate on the CFX96 real-time system (Bio-Rad) using a fast-cycling program: initial enzyme activation for 2 min at 95°C, followed by 40 cycles of denaturation for 5 sec at 95°C and an annealing/extension for 30 sec at 60°C. Statistical significance was determined using a unpaired two-tailed Welch's *t*-test across three biological replicates for each time point.

### Data sets

All data sets analyzed in this study are publicly available. The bulk RNA-seq data were obtained from NCBI Gene Expression Omnibus (GEO; https://www.ncbi.nlm.nih.gov/geo/) under accession numbers GSE85331 (Liu et al. 2017) and GSE106688 (Bertero et al. 2019). The single-cell RNA-seq data set (scRNA-seq) for hiPSC-derived cardiomyocyte differentiation is available in the ArrayExpress database (https://www.ebi.ac.uk/biostudies/arrayexpress) under accession number E-MTAB-6268. The fetal human heart scRNA-seq is available in GEO under accession number GSE106118.

## Data access

All raw and processed sequencing data generated in this study have been submitted to the NCBI Gene Expression Omnibus (GEO; https://www.ncbi.nlm.nih.gov/geo/) under accession number GSE274653.

## Supplemental Material

Supplement 1

Supplement 2
