# Supplementary material for: Dynamics and consequences of differential RNA isoform production during cardiomyocyte fate determination and early-stage maturation
Source: Genome Res. 2026 May;36(5):903–15. doi: 10.1101/gr.281150.125 (PMC13445713; doi:10.1101/gr.281150.125)
Supplement: Supplementary file 1 [file Supplemental_Figures.docx]

**Supplemental Figures**

**Dynamics and consequences of differential RNA isoform production during cardiomyocyte fate determination and early-stage maturation**

Joanna Delimata-Raczek^1^, Natalia Koralewska^1^, Bart Krist^1^, Magdalena Rakoczy^1^, Marek Figlerowicz^1^, Ireneusz Stolarek^1*^

^1^ Institute of Bioorganic Chemistry Polish Academy of Sciences, Noskowskiego 12/14, 61-704 Poznan, Poland

* Correspondence and requests for materials should be addressed to:

Dr. Ireneusz Stolarek

email: istolarek@ibch.poznan.pl

**Supplemental Figure S1.** Replication results of the identified events associated with DTA. The numbers in circles denote DTA-displaying genes identified in each dataset with DTUrtle software. The final number of identified DTA-displaying genes is shown in the triangle, as these genes were identified in at least two datasets by two computational methods: DTUrtle and IsoformAnalyzeR.

**Supplemental Figure S2.** Illustration of the alternative splicing/transcription events associated with DTA identified in bulk RNA-seq on the example of Liu *et al*. dataset (Liu et al. 2017) for genes selected for validation by qPCR: **(A)** *GCNT2***, (B)** *ATP2A2***, (C)** *TPM2***-C)**.

**Supplemental Figure S3.** Statistical significance test results for the alternative splicing/transcription events associated with DTA.

**Supplemental Figure S4.** GSEA for genes displaying DTA between D4 and D30 of iPSC-to-iCM differentiation with the RNA isoform switch consequence regarding coding potential change. **(A)** all genes displaying coding potential change, **(B)** genes with downregulation of RNA isoform with coding potential, **(C)** genes with upregulation of RNA isoform with coding potential.

**Supplemental Figure S5.** Representative alternative splicing and transcription events associated with DTA. **(A-D)** Events identified in canonical cardiomyocyte genes. **(E-H)** Events identified in non-cardiomyocyte-specific genes.

**Supplemental Figure S6.** scRNA-seq data of iPSC-to-iCM differentiation at D5, D15 and D30. **(A)** UMAP embedding colored by differentiation stage. **(B)** UMAP embedding colored by Seurat cell cluster. **(C-E)** Feature plots showing the expression of contractile cell markers: **(C)** *TTN*, **(D)** *MYH6*, **(E)** *MYH7*. Feature plot showing the expression of non-contractile cell marker, *DCN*.

**Supplemental Figure S7.** scRNA-seq data of *ex vivo* human fetal heart tissue. **(A)** UMAP embedding colored by Seurat cell clusters. **(B-D)** Feature plots showing expression of contractile cell markers: **(B)** *TTN*, **(C)** *MYH6*, **(D)** *MYH7*. **(E)** Feature plot showing the expression of non-contractile cell marker, *DCN*.

**Supplemental Figure S8.** Gene list similarity network of biological processes enriched among DGE^-^/DTA^+^ (orange), DGE^+^/DTA^+^ (green), and DGE^+^/DTA^-^ (blue) gene sets. Node color indicates the gene regulation mode, node size is proportional to the number of genes associated with each biological process. Edges connect nodes that share at least 30% of genes.

**Supplemental Figure S9.** Gene list similarity heatmap of GO Biological Process terms enriched in genes exhibiting DTA between D4 and D30.

**Supplemental Figure S10.** Distribution of contractile and non-contractile cells across gene expression regulation modes. **(A)** Stacked bar plot showing cell type distribution within each HD cluster, categorized by DGE and DTA status. **(B)** Proportions of contractile (red) versus non-contractile (blue) cells across specific DGE and DTA conditions (DGE^-^/DTA^+^, DGE^+^/DTA^+^, and DGE^+^/DTA^-^). Sample sizes (*n*) are indicated above each bar.The numbers above the bars indicate the sample sizes (n) for each condition.

**Supplemental Figure S11.** Enrichment of RBP binding sites in DTA-associated transcripts (D4 vs. D30). **(A)** Number of DeepBIND-predicted binding sites in RNA isoforms for genes with significant DTA. **(B)** Distribution of corresponding DeepBIND scores.
